# The impact of human-AI collaboration types on consumer evaluation and usage intention: a perspective of responsibility attribution

**DOI:** 10.3389/fpsyg.2023.1277861

**Published:** 2023-10-30

**Authors:** Beibei Yue, Hu Li

**Affiliations:** ^1^School of Business, Qingdao University, Qingdao, Shandong, China; ^2^School of Business, Qingdao University, Qingdao, Shandong, China

**Keywords:** artificial intelligence, human-AI collaboration, outcome expectation, evaluation, usage intention, responsibility attribution, algorithm transparency

## Abstract

Despite the widespread availability of artificial intelligence (AI) products and services, consumer evaluations and adoption intentions have not met expectations. Existing research mainly focuses on AI’s instrumental attributes from the consumer perspective, along with negative impacts of AI failures on evaluations and willingness to use. However, research is lacking on AI as a collaborative agent, investigating the impact of human-AI collaboration on AI acceptance under different outcome expectations. This study examines the interactive effects of human-AI collaboration types (AI-dominant vs. AI-assisted) and outcome expectations (positive vs. negative) on AI product evaluations and usage willingness, along with the underlying mechanisms, from a human-AI relationship perspective. It also investigates the moderating role of algorithm transparency in these effects. Using three online experiments with analysis of variance and bootstrap methods, the study validates these interactive mechanisms, revealing the mediating role of attribution and moderating role of algorithm transparency. Experiment 1 confirms the interactive effects of human-AI collaboration types and outcome expectations on consumer evaluations and usage willingness. Under positive outcome expectations, consumers evaluate and express willingness to use AI-dominant intelligent vehicles with autonomous driving capabilities higher than those with emergency evasion capabilities (AI-assisted). However, under negative outcome expectations, consumers rate autonomous driving capabilities lower compared to emergency evasion capabilities. Experiment 2 examines the mediating role of attribution through ChatGPT’s dominant or assisting role under different outcome expectations. Experiment 3 uses a clinical decision-making system to study algorithm transparency’s moderating role, showing higher transparency improves evaluations and willingness to use AI products and services under negative outcome expectations. Theoretically, this study advances consumer behavior research by exploring the human-AI relationship within artificial intelligence, enhancing understanding of consumer acceptance variations. Practically, it offers insights for better integrating AI products and services into the market.

## 1. Introduction

While AI products and services have rapidly integrated into consumer lives and led a new wave of product transformation, consumer acceptance of different types of AI products and services has not met expectations. Take autonomous driving technology as an example, despite its significantly lower failure rate compared to accidents caused by human operation, consumers still maintain a cautious stance toward self-driving cars, with varying opinions. Conversely, emergency evasion technology applied in the same automotive driving context has garnered high praise and enthusiastic adoption from users (Peng et al., 2022, 00187208221113448). One contributing factor to this phenomenon could be the negative media coverage that has diminished consumer expectations of AI products and services (Shank et al., 2019, 648–663). Most of the existing research primarily explains this phenomenon from the perspectives of consumers as users and AI technology as a tool. For instance, the user perspective primarily explores the impact of factors such as consumer demand, engagement, and usage feedback on the adoption of AI products and services (Collier and Sherrell, 2010, 490–509; Park and McKilligan, 2018, 725–740). The tool perspective examines the influence of different technological conditions on product and service effectiveness, such as technological availability and usability, task fit, and technological advantages (Cuddy et al., 2008, 61–149; Lee et al., 2012, 319–326; Kaur and Rampersad, 2018, 87–96). However, as AI systems have demonstrated significant intelligence and autonomy across various domains, AI is transitioning from a tool role to a “partner” role. Humans and AI can collaborate closely to achieve common goals, and this form of human-AI collaboration is a crucial application of AI technology (Mao et al., 2019, Article 237). There has been limited research from the perspective of human-AI relationships discussing how human-AI collaboration may differentially affect consumers’ adoption of AI products and services. As the dynamics of human-AI relationships evolve, it is essential to consider the current positioning of humans and their levels of acceptance of AI. This study posits that consumer acceptance of AI products and services may be influenced both by their expectations of product and service outcomes and by their perceptions and attitudes toward AI autonomy. With the continuous improvement in AI autonomy, AI is transitioning from a mere tool to a “partner” capable of collaborative interaction with users. Balancing the human-AI relationship during this collaborative process stands as a crucial determinant of AI acceptance (Mao et al., 2019, Article 237). Furthermore, a natural algorithmic fear among the public toward AI technology impacts consumer acceptance and adoption of AI products and services (Adadi and Berrada, 2018, 52,138–52,160). According to research, if users are unable to comprehend the workings and decision-making processes of artificial intelligence, it can adversely affect collaborative effectiveness and user experience (Stubbs et al., 2007, 42–50). So, it is worth considering whether improving algorithm transparency can enhance people’s acceptance of AI.

In summary, the objective of this study is to investigate the interactive effects of human-AI collaboration types and outcome expectations on consumer evaluations and adoption of AI products and services through a mediated moderation model of responsibility attribution. Furthermore, this research aims to explore the moderating role of algorithm transparency in this interactive relationship. By delving into the differentiated reasons behind consumer acceptance levels of AI products and services, this study seeks to offer valuable guidance and support in the realm of consumer behavior, thus facilitating the promotion and advancement of AI products and services in the market.

## 2. Research review

### 2.1. Artificial intelligence and human-AI collaboration

Artificial Intelligence (AI), as a computer technology capable of simulating human intelligence, utilizes methods such as machine learning, deep learning, knowledge representation, and reasoning to perform various tasks and address complex problems (Franke et al., 2019, 456–467). AI possesses superior information processing and computational analysis capabilities compared to humans, enabling the completion of tasks beyond human capacity. Human-AI Collaboration refers to a form of AI application in which humans and AI collaborate closely to achieve a shared objective (Maddikunta et al., 2022). It encompasses the combined efforts of AI and humans in terms of their collaborative agency, building upon the foundation of human-computer interaction (Abbass, 2019, 159–171). Thus, Human-AI Collaboration capitalizes on human cognitive abilities and AI computational capabilities, resulting in more efficient work processes and improved outcomes (Abbass, 2019, 159–171). Existing research indicates that within the medical field, AI-supported systems can assist doctors in making more efficient and accurate diagnoses during Human-AI collaboration (Reverberi et al., 2022, 14952). In design-related domains, generative AI can balance creativity and efficiency in the creative process, facilitating rapid product innovation (Louie et al., 2020, 1–13).

During the process of Human-AI Collaboration, AI can “extend” human capabilities through its efficient computational power and precise execution (Abbass, 2019, 159–171). Concurrently, humans can compensate for AI’s shortcomings by contributing emotional perception and subjective judgment capabilities that AI lacks (Sundar, 2020, 74–88). Existing research acknowledges the superiority of Human-AI Collaboration over either human or AI alone. For instance, Lai et al. (2022) found that compared to individual human or AI review of content and comments on social media platforms, a Human-AI collaboration approach can synergize their strengths, enhancing accuracy and credibility in content review. However, the extent to which Human-AI Collaboration should be pursued and how to balance the relationship between AI and humans within this context remains unclear. Scholars in the field of computer science focus on maximizing AI autonomy in Human-AI Collaboration, striving for AI’s independent decision-making capability (Rahwan et al., 2019, 477–486). Nevertheless, research in consumer behavior suggests that consumer acceptance of AI products and services is not always positively correlated with AI autonomy (Serenko, 2007, 293–303). In fact, consumers might reject highly autonomous AI products and services due to perceived threats posed by AI (Oh et al., 2018). Relevant studies highlight that users’ sense of control during Human-AI Collaboration impacts their trust and understanding of AI products and services, subsequently influencing their adoption (Westphal et al., 2023, 107714). Therefore, considering the influence of AI autonomy or control in collaborative processes on consumer acceptance of AI products and services becomes crucial for understanding consumer preferences.

This study, based on Serenko’s research (Serenko, 2007, 293–303), categorizes Human-AI Collaboration into AI-Dominant and AI-Assisted types, based on the degree of AI autonomy in the collaborative process. AI-Dominant refers to scenarios where AI is the task executor and responsible for task completion, with humans primarily initiating tasks and overseeing processes. AI-Assisted entails humans being task executors responsible for task completion, with AI playing a supporting role during the task completion process.

### 2.2. Research on the evaluation and usage behavior of AI products and services

Consumer evaluations and adoption behaviors of AI products and services exhibit significant differences (Scherer et al., 2019, 13–35). Existing research has predominantly interpreted this phenomenon from user-centric and tool-centric perspectives. The user-centric approach aims to enhance user experience to meet consumer demands for AI products and services. Research has revealed that consumer psychological traits have differentiated impacts on the evaluation of AI products and services. Factors such as varying expectations of service robots, curiosity about AI, and attitudes (including biases and aversions) influence the evaluation and usage intentions of AI products and services (Crolic et al., 2022, 132–148; Zhang et al., 2022, 2,171–2,183; Wang et al., 2023, 177–193). The tool-centric perspective focuses on the differences in the influence of technical features of AI products and services on consumers. For instance, Yang et al. (2020, 1–13) argue that uncertainties in the capabilities of intelligent systems, transparency of decision-making processes, and complexity of output results can lead consumers to maintain a cautious attitude toward AI products and services. Research has also indicated that users doubt the accuracy of AI involvement in content moderation on social platforms due to inherent emotional capabilities of AI technology (Sundar et al., 2015, 47–86). Furthermore, the ease of use of AI products and services (i.e., whether the technology is overwhelming) also contributes to differences in consumer evaluations and adoption behaviors (Kim et al., 2022, 79–96). Addressing these evaluation differences from various perspectives, some research has further considered the impact of interaction quality on consumer evaluations of AI products and services from the perspective of human-AI interaction, suggesting that effective user interfaces and interaction design can enhance consumer engagement and satisfaction (Rahwan et al., 2019, 477–486). However, most studies from a human-computer interaction perspective still treat AI as a tool and rarely consider AI as a subject, neglecting the role of AI as a principal in collaboration with humans. Shank et al. (2019, 648–663) suggest that focusing on the principal role of AI in human-AI collaboration, and reconsidering the roles and division of labor between humans and AI, can better address diverse consumer needs. Song et al. (2022, 102900) further emphasizes that the key to balancing the relationship between AI and humans in services lies in accurately defining the role of AI in human-machine collaboration. Therefore, exploring the role differentiation between AI and humans in Human-AI Collaboration, whether as a principal or an assistant, may be the key to interpreting differentiated consumer evaluations and adoption of AI products and services.

This study is situated precisely within the context of role differentiation between AI and humans in Human-AI Collaboration, investigating the impact of Human-AI Collaboration types on consumer evaluations and usage of AI products and services. This inquiry aims to further elucidate the reasons behind differentiated consumer evaluations and usage intentions. As consumer evaluations of AI products and services transition from evaluating tools to evaluating principals, it becomes essential to consider the allocation of responsibilities between humans and AI in the collaborative process. However, existing research often only acknowledges AI’s responsibility and attributes negative outcomes to AI when they occur, subsequently leading to negative evaluations of AI (van der Woerdt and Haselager, 2019, 93–100), thus overlooking the attribution effect of positive outcomes to AI’s contributions and its impact on consumer evaluations and usage of AI products and services. Whether consumers attribute success to AI and subsequently enhance their evaluation and adoption behaviors toward AI products and services under positive outcomes remains uncertain. Thus, based on attribution theory, this study aims to explore the interactive influence of Human-AI Collaboration types and outcome expectations on consumer evaluations and usage behaviors of AI products and services.

## 3. Research hypotheses

### 3.1. Interaction between human-AI collaboration types and outcome expectations

Outcome Expectations refers to an individual’s envisaged or inferred outcomes arising from a particular action (Strathman et al., 1994, 742–752). Positive outcome expectation involves an individual’s optimistic projections or inferences regarding the potential outcomes, while negative outcome expectation entails a predisposition toward pessimistic expectations. In the context of consumers’ perceptions toward products and services, positive outcome expectation encompasses the notion that the acquisition and utilization of specific products or services will satisfy their needs and engender positive experiential outcomes. This expectation subsequently influences consumers’ recognition and embrace of the products and services. Conversely, negative outcome expectation entails consumers’ apprehension of adverse effects or detrimental repercussions that could arise from acquiring and employing particular products or services. This apprehension encompasses concerns encompassing factors such as quality, experiential dimensions, and even safety considerations. These negative expectations may instigate doubt and skepticism concerning the efficacy of products and services, leading to a reduction in the inclination to purchase and use them (Baumeister et al., 2001, 323–370).

Existing research in the domain of consumer behavior underscores that outcome expectations play a pivotal role in the decision-making process, influencing consumers’ subjective assessments of products and services during the information gathering and evaluation phases. This, in turn, substantially impacts their eventual purchasing and usage behaviors (Gu et al., 2010, 200–206). Drawing from the Technology Acceptance Model, it is elucidated that when consumers engage with and acquire products and services that incorporate novel technologies, they weigh the perceived utility of the technology and the ease of its utilization as crucial benchmarks for adoption and acceptance (Venkatesh et al., 2003, 425–478). For instance, the accessibility and user-friendliness of AI technology are instrumental in shaping consumers’ evaluations and behavioral inclinations toward AI products and services (Cuddy et al., 2008, 61–149).

Within the framework of consumers’ engagement with AI products and services, the potential for errors to stem from either human or AI components is recognized as consequential to the attainment of desired outcomes. Positive outcome expectation signifies that both human and AI agents within the Human-AI Collaboration context have been contributors to the manifestation of favorable results. Furthermore, the Halo Effect posits that consumers may exhibit favorable sentiments toward AI entities within collaborative frameworks due to positive outcome expectations. Therefore, in instances where consumers harbor positive expectations regarding the acquisition or utilization of AI products and services, it implies their attribution of value to the role played by AI products and services in facilitating positive outcomes. This association is accentuated by the higher impact of AI technology on positive outcomes within AI-Dominant products and services in comparison to AI-Assisted variants. Conversely, in cases where consumers hold negative outcome expectations toward AI products and services, it signifies their perception of uncertainties and risks associated with the effectiveness of these offerings. This skepticism is accompanied by a cautious attitude toward the reliability of AI technology, alongside the classification of AI as a potential source of ambiguity and risk. Given the heightened involvement of AI technology within AI-Dominant Human-AI Collaborations as opposed to AI-Assisted models, consumers may associate AI-Dominant products and services with elevated levels of uncertainty and risk. Aligned with the tenets of Prospect Theory, individuals are generally inclined to evade uncertain risks due to a prevalent aversion to losses. Consequently, within scenarios marked by negative outcome expectation, AI-Dominant models are anticipated to dampen consumers’ evaluations and their willingness to employ AI products and services in comparison to AI-Assisted counterparts. Drawing from the aforementioned, the formulated hypotheses are as follows:

*Hypothesis* 1 (H1): The classification of Human-AI Collaboration types (AI-Dominant vs. AI-Assisted) interacts substantially with the nature of outcome expectations (positive vs. negative) to exert an influence on consumers’ evaluations and their propensities to use AI products.

*Hypothesis* 1a (H1a): Within contexts characterized by positive outcome expectations, consumers are anticipated to exhibit enhanced evaluations and heightened inclinations to utilize AI-Dominant products and services relative to AI-Assisted alternatives.

*Hypothesis* 1b (H1b): In scenarios marked by negative outcome expectations, consumers’ evaluations and their propensity to employ AI-Dominant products and services are anticipated to be comparatively diminished in comparison to AI-Assisted variants.

### 3.2. Mediating role of attribution of responsibility

Attribution theory posits that individuals possess an inherent motivation to comprehend and explain the causes behind events occurring in their environment. In this context, attribution of responsibility pertains to the cognitive processes individuals employ when assessing and attributing reasons for events or outcomes. This process encompasses two distinct types: internal attribution and external attribution (Heider, 1958). Internal attribution refers to attributing events or outcomes to one’s own abilities, attitudes, or personality traits, while external attribution attributes the results of events or actions to external factors like the environment, opportunities, or the influence of others. Attribution represents a subjective interpretation by individuals of the causes underlying events and behaviors, and while it may deviate from objective reality, it often wields a greater influence on individual behavior than factual and objective causes (Jenkins et al., 2014, 17–33).

Currently, within the realm of marketing, there is considerable research attention directed toward issues related to the attribution of responsibility in service outcomes (Albrecht et al., 2017, 188–203). In cases of negative service outcomes, the attribution of responsibility may lead to negative consumer evaluations of services, decreased satisfaction levels, and a reduction in purchase intent (Choi and Mattila, 2008, 24–30; Kalamas et al., 2008, 813–824). Conversely, positive service outcomes can enhance consumer satisfaction by acknowledging the contributions that led to these positive outcomes. In the context of AI research within marketing, scholars have primarily focused on AI’s responsibility in negative events, while paying limited attention to how AI’s contributions in positive events influence consumer behavior (Shank et al., 2019, 648–663).

Given that in AI-dominant human-AI collaboration, AI is the executor of tasks, whereas in AI-assisted collaboration, AI plays a supporting role in task completion, the influence of AI on task completion is higher in AI-dominant scenarios compared to AI-assisted ones. When consumers hold positive expectations for AI products and services, they assess and judge the factors that contribute to the positive outcomes. As AI’s contribution to positive outcomes is higher in AI-dominant collaborations compared to AI-assisted ones, consumers attribute greater contribution to AI in AI-dominant scenarios. Consequently, they evaluate and rate AI-dominant products and services more positively than their AI-assisted counterparts. Conversely, in cases where consumers anticipate negative outcomes from AI products and services, they similarly evaluate and judge the reasons for these negative outcomes. Prior research has underscored that users tend to attribute blame to AI when negative outcomes occur while using AI products and services (Serenko, 2007, 293–303). Because AI functions as the primary executor in AI-dominant collaborations and assumes a supportive role in AI-assisted collaborations, consumers attribute higher failure to AI in AI-dominant scenarios when negative outcome expectation exists. This, in turn, outcomes in lower evaluation and utilization intent for AI-dominant products and services compared to their AI-assisted counterparts. As a consequence, this study presents the following hypothesis:

*H*2: Attribution of responsibility plays a mediating role in the process of the interactive influence of human-AI collaboration type and outcome expectation on consumer evaluation and utilization intent.

### 3.3. Role regulatory role of algorithm transparency

In the field of artificial intelligence, transparency refers to the extent to which an intelligent system can provide insight into its internal workings, encompassing two distinct aspects: interpretability and readability (Rosenfeld and Richardson, 2019, 673–705). Interpretability pertains to the user’s ability to comprehend the operational mechanisms of an AI system, while readability pertains to the user’s ability to understand the outcomes produced by the AI system. Algorithmic transparency signifies the degree to which individuals can comprehend, interpret, and predict the operational processes and outcomes of AI algorithms (Lipton, 2018, 36–43). Transparent algorithms facilitate users’ understanding of the operational dynamics and decision-making processes of AI systems, thereby fostering better comprehension and trust in the algorithms’ decisions. The absence of algorithmic transparency can potentially lead to negative impacts on user experience (Ribeiro et al., 2016, 1,135–1,144). These negative effects might remain latent when AI aligns with user expectations. For instance, users may trust and employ AI products that fulfill their needs, even if they lack an understanding of the AI’s operational processes (Lee and See, 2004, 50–80; West, 2018, 4,366–4,383). However, in cases where discrepancies arise during the use of AI products, especially when outcomes deviate from user expectations, the lack of understanding of algorithmic decision-making could lead to skepticism, distrust, fear, and even rejection of AI (Robinette et al., 2017, 425–436). For instance, when a self-driving car adhering to traffic rules is involved in an accident, uninformed consumers might attribute blame to the self-driving car and subsequently provide a negative evaluation (Hong et al., 2022, 102–102).

Algorithmic transparency can significantly influence consumers’ evaluations and use of AI products and services. High algorithmic transparency can enhance consumers’ perception of autonomy, subsequently augmenting their acceptance of AI-recommended products (Basso et al., 2016, 14–25). When consumers hold positive expectations regarding the purchase and use of AI products and services, the presence or absence of high algorithmic transparency does not significantly affect their evaluations and usage intentions. This is primarily due to the halo effect associated with positive outcomes, whereby consumers are inclined to hold favorable evaluations and intentions toward both AI-dominant and AI-assisted products and services, regardless of whether they comprehend the decision-making processes and operational mechanisms of AI. However, in scenarios characterized by negative expectations regarding the outcomes of AI products and services, the level of algorithmic transparency can influence consumers’ evaluations. Specifically, under conditions of low algorithmic transparency, consumers’ lack of understanding regarding AI’s operational processes and outcomes might lead them to instinctively associate unfavorable outcomes with AI, thereby questioning AI’s reliability. As AI-dominant products and services involve higher AI influence on outcomes compared to AI-assisted ones, consumers’ evaluations and usage intentions toward AI-dominant products and services are likely to be lower. Conversely, in scenarios characterized by high algorithmic transparency, users’ improved comprehension of algorithmic workings and decision processes enables a clearer and more objective perception of AI’s reliability. This enhanced understanding facilitates users’ appreciation of the limitations of artificial intelligence algorithms and consequently improves their evaluations and intentions to use AI products and services. Building upon these observations, this study presents the following hypotheses:

*H*3: In scenarios of negative outcome expectations, high algorithmic transparency significantly enhances consumers’ evaluations and usage intentions toward AI products and services compared to low algorithmic transparency.

*H*3a: In scenarios of positive outcome expectations, there is no significant difference in consumers’ evaluations and usage intentions toward AI products and services between high and low algorithmic transparency conditions.

*H*3b: In scenarios of negative outcome expectations, high algorithmic transparency enhances consumers’ evaluations and usage intentions toward AI products and services compared to low algorithmic transparency.

**Figure 1:**
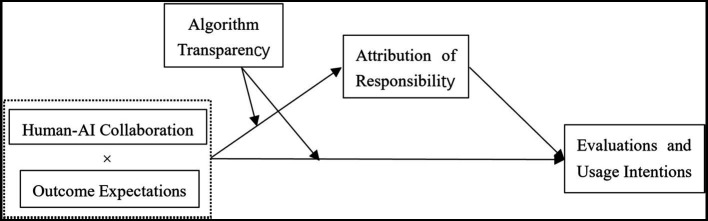
Variable analysis path model.

Based on the above hypotheses, our variable analysis path model is shown in Figure 1.

## 4. Experimental results and analysis

### 4.1. Research design

This study employed three experiments to test hypotheses H1-H3. Experiment 1 utilized a 2×2 between-subjects design involving the factors of Human-AI Collaboration Type (AI Dominant vs. AI Assistant) and Outcome Expectancy (Positive vs. Negative) to examine participants’ evaluations and usage intentions toward AI products and services. Similarly, Experiment 2 also employed a 2×2 between-subjects design, incorporating measurements of responsibility attribution to analyze the mediating effect of responsibility attribution. Experiment 3 adopted a 2×2×2 between-subjects design involving Human-AI Collaboration Type (AI Dominant vs. AI Assistant), Outcome Expectancy (Positive vs. Negative), and Algorithm Transparency (High vs. Low), manipulating algorithm transparency to explore its moderating effect.

### 4.2. Experiment 1

The primary objective of Experiment 1 was to investigate consumers’ evaluations and usage intentions toward different types of AI products and services in varying outcome expectancy scenarios. Specifically, the aim was to validate Hypothesis H1, which posits that consumers’ evaluations and usage intentions will be influenced by the interaction between outcome expectancy and the type of human-AI collaboration.

#### 4.2.1. Design and participants

Experiment 1 employed a 2 × 2 between-subjects design involving the manipulation of Human-AI Collaboration Type (AI Dominant vs. AI Assistant) and Outcome Expectancy (Positive vs. Negative). Participants were randomly assigned to one of four experimental groups based on the combination of these variables. The study aimed to assess participants’ evaluations and usage intentions concerning intelligent products. In this experiment, intelligent automobiles were utilized as the subject material. The automatic driving and emergency evasion functions of the smart car were manipulated to induce AI Dominant and AI Assistant scenarios.

To obtain a generalizable result, we recruited participants on the Credamo platform under the guise of a market research study on smart car usage, without imposing any restrictions on the participants. A total of 199 participants were initially recruited. After eliminating 23 incomplete or inadequate responses, the final valid sample consisted of 176 participants. Of these, 105 were female (59.7%) and 71 were male (40.3%), spanning an age range of 18 to 60 years. All participants volunteered to take part, and the design was approved by the appropriate ethics review board. The participants were evenly distributed across four groups: AI Dominant—Positive Outcome Expectancy (42 participants), AI Dominant—Negative Outcome Expectancy (47 participants), AI Assistant—Positive Outcome Expectancy (41 participants), and AI Assistant—Negative Outcome Expectancy (46 participants). The groups exhibited no significant differences in terms of gender and age. This meticulous experimental design ensured that potential confounding variables were controlled, allowing for a rigorous investigation into the effects of Human-AI Collaboration Type and Outcome Expectancy on participants’ evaluations and usage intentions of intelligent products.

#### 4.2.2. Experimental procedure

During the experiment, participants were initially presented with a description of a car purchasing scenario. They were informed that “smart cars” referred to vehicles equipped with advanced information technology and artificial intelligence, enabling features like autonomous driving and intelligent safety functions.

Subsequently, participants were immersed in a typical car-buying situation. In this context, participants were led to imagine themselves recently considering the purchase of a smart car for household use. They were then exposed to information about a specific smart car model as part of their information-gathering process.

To manipulate participants’ perception of human-AI collaboration types, a method similar to Serenko (2007, 293–303) was adapted. Participants were randomly assigned to either the AI Dominant or AI Assistant group, denoted as Group AI-D and Group AI-A, respectively. The description provided to Group AI-D emphasized the automatic driving feature of the smart car, highlighting its ability to drive for longer distances without human intervention. In contrast, Group AI-A was presented with a description focusing on the emergency avoidance feature, illustrating how the smart car could act promptly in unexpected situations when the driver could not react in time.

A perception assessment, based on the approach by Molina et al. (2022, zmac010) followed this manipulation. The assessment included items such as “This driving process is controlled by a person/artificial intelligence.” and “This driving process is completed by a person/artificial intelligence.” Participants rated these items on a 7-point scale, with 1 indicating human control and 7 indicating AI control.

After comprehending the smart car’s functionalities, participants were randomly allocated to different Outcome Expectancy groups. Consumer expectations of product outcomes are influenced by external factors such as online reviews, word-of-mouth, and advertising appeals (Grunewald and Kräkel, 2017, 91–113). In the Positive Outcome Expectancy group, participants were exposed to external information suggesting that owners of this smart car brand successfully reached their destinations every time they used the autonomous driving/emergency avoidance function. In the Negative Outcome Expectancy group, participants were exposed to external information about an incident where a smart car owner encountered a traffic accident while utilizing the autonomous driving/emergency avoidance function.

Subsequently, participants were asked to evaluate their perceived likelihood that “When using the intelligent mode of this car, the owner will arrive at their destination smoothly.” This evaluation was also rated on a 7-point scale, where 1 represented low likelihood and 7 indicated high likelihood.

After reading through the experimental scenario, participants were required to respond to questions related to their evaluations of the smart car and their willingness to use it, inspired by the approach employed by Venkatesh et al. (2003, 425–478). The evaluation section consisted of three items: “The artificial intelligence technology used in this smart car is good,” “I consider the artificial intelligence technology to be useful,” and “I like using this artificial intelligence technology.” Similarly, the section regarding usage intentions also comprised four items: “I am willing to collect information about the smart car,” “I am willing to recommend this smart car to others,” “I am prepared to use this smart car in the near future,” and “If needed, I am willing to purchase this smart car.” All items were rated on a 7-point Likert scale, where 1 indicated “strongly disagree” and 7 indicated “strongly agree.”

#### 4.2.3. Experimental results

(1) Manipulation check

The results of the independent samples t-tests revealed significant differences in participants’ perceptions of AI’s role based on the manipulation of human-AI collaboration types. Specifically, participants in the AI-dominant group (M_AI-dominant_ = 6.354, SD = 0.683, *n* = 89) demonstrated significantly higher perceptions of AI’s influence compared to those in the AI-assistive group (M_AI-assistive_ = 4.086, SD = 1.918, *n* = 87; *t* = 10.491, *p* < 0.001, Cohen’s *d* = 1.575). This indicates the successful manipulation of human-AI collaboration types.

Additionally, the independent samples *t*-test results illustrated substantial differences in perceived task success levels between the positive outcome expectancy group and the negative outcome expectancy group. Participants in the positive outcome expectancy group (M_positive_ = 6.39, SD = 0.730, *n* = 89) had significantly higher perceptions of task success compared to those in the negative outcome expectancy group (M_negative_ = 3.47, SD = 1.711, *n* = 87; *t* = 14.384, *p* < 0.001, Cohen’s *d* = 2.220). This confirms the effectiveness of the outcome expectancy manipulation.

(2) Hypothesis testing

Interaction effects analysis. The results of the two-way ANOVA revealed a significant interaction between human-AI collaboration types and outcome expectancies on consumer evaluations [*F*(1, 172) = 23.731, *p* < 0.001, η^2^ = 0.121] as well as usage intentions [F(1, 172) = 26.910, *p* < 0.001, η^2^ = 0.135], thus confirming the validity of Hypothesis 1. Further analysis of the simple effects showed that under positive outcome expectancy, the AI-dominant group exhibited higher evaluations [M_AI-dominant_ = 6.103, SD = 0.463 vs. M_AI-assistive_ = 5.862, SD = 0.628; F(1, 172) = 3.714, *p* = 0.061] and usage intentions [M_AI-dominant_ = 6.137, SD = 0.463 vs. M_AI-assistive_ = 5.738, SD = 0.575; F(1, 172) = 15.385, *p* < 0.001] than the AI-assistive group, confirming Hypothesis 1a.

**Figure 2:**
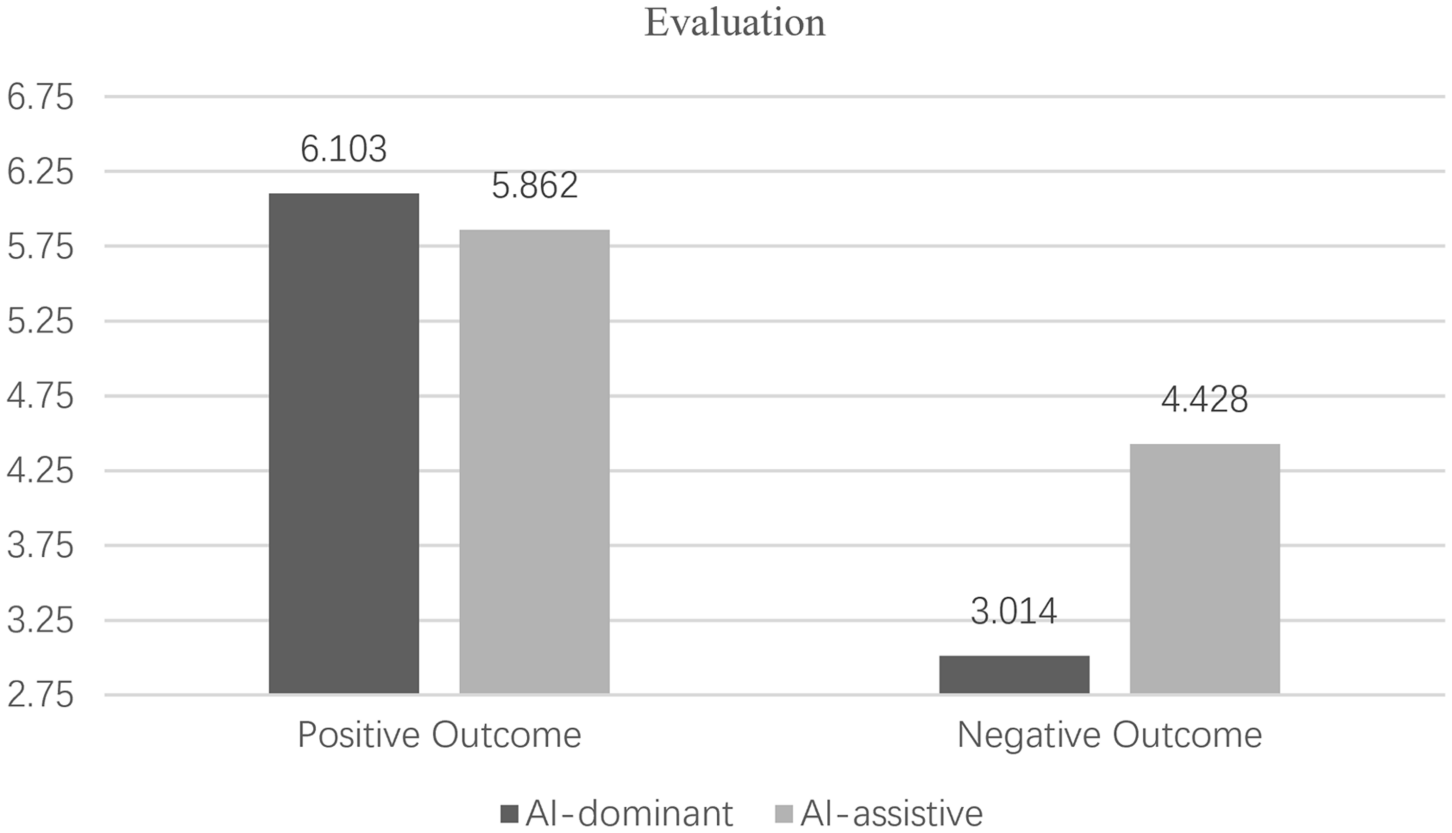
Interactive effect of human-AI collaboration and outcome expectations on evaluation.

**Figure 3:**
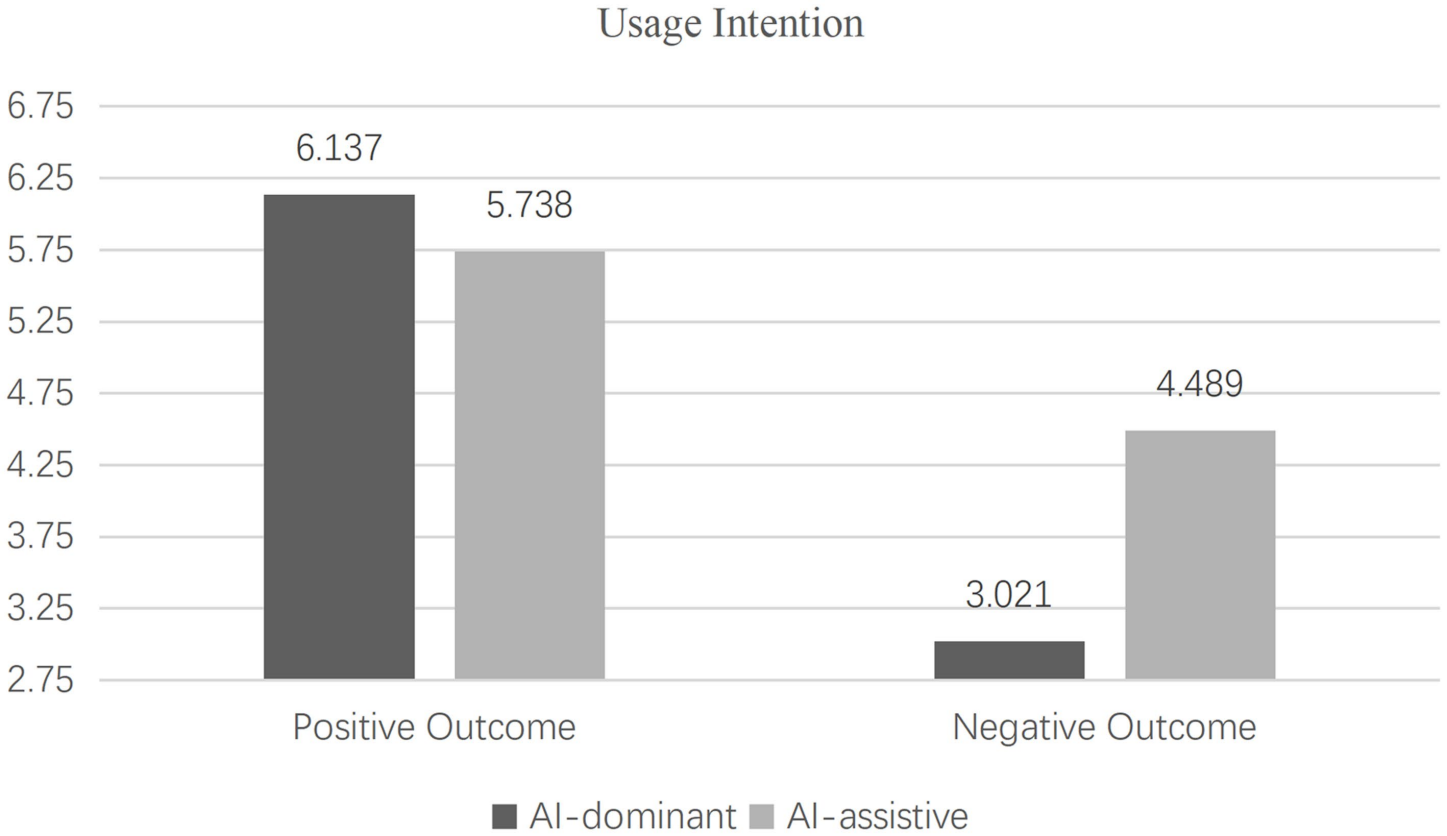
Interactive effect of human-AI collaboration and outcome expectations on usage intention.

Conversely, under negative outcome expectancy, the AI-dominant group demonstrated lower evaluations [M_AI-dominant_ = 3.014, SD = 1.483 vs. M_AI-assistive_ = 4.428, SD = 1.429; F(1, 172) = 25.301, *p* < 0.001] and usage intentions [M_AI-dominant_ = 3.021, SD = 1.609 vs. M_AI-assistive_ = 4.489, SD = 1.514; F(1, 172) = 19.918, *p* < 0.001] compared to the AI-assistive group, validating Hypothesis 1b. The specific results are dissected in Figures 2, 3.

#### 4.2.4. Discussion of experiment 1

Results from Experiment 1 highlight that varying outcome expectancies influence consumer evaluations and usage intentions toward AI products and services across different human-AI collaboration types. Notably, AI-dominant products and services are more susceptible to the effects of outcome expectancies when compared to their AI-assistive counterparts. To further investigate the interaction effects and their underlying mechanisms, Experiment 2 introduces responsibility attribution as a mediating mechanism, while enhancing the robustness of results through the substitution of AI products.

### 4.3. Experiment 2

The purpose of Experiment 2 is to examine Hypothesis H2, which posits that responsibility attribution mediates the interactive effects of human-AI collaboration types and outcome expectancies on consumer evaluations and usage intentions. Additionally, Experiment 2 seeks to further validate Hypothesis H1.

#### 4.3.1. Design and participants

Experiment 2 employed a 2 × 2 between-subjects design to investigate the mediating role of responsibility attribution in the interactive effects between human-AI collaboration types (AI-dominant vs. AI-assisted) and outcome expectancies (positive vs. negative) on consumer evaluations and usage intentions. The study focused on the widely recognized AI system ChatGPT, given its practical applications in everyday work and learning contexts as a natural language processing tool.

Under the pretense of conducting a product survey on the effectiveness of ChatGPT usage for an internet company, 192 participants were recruited through the Credamo platform. After excluding 23 inconsistent or inadequate responses, a total of 169 valid participants remained. Of these, 93 were female (55.1%) and 76 were male (44.9%), with ages ranging from 18 to 60 years. All participants volunteered to take part, and the design was approved by the appropriate ethics review board. Participants were randomly assigned to four distinct groups: AI-dominant with positive outcome expectancy (44 participants), AI-dominant with negative outcome expectancy (40 participants), AI-assisted with positive outcome expectancy (45 participants), and AI-assisted with negative outcome expectancy (40 participants). No significant differences were observed among the four groups in terms of gender and age.

#### 4.3.2. Experimental procedure

Initially, participants were guided into a simulated work or learning scenario that involved frequent submission of written materials, representative of a typical context for ChatGPT application. Subsequently, participants were introduced to ChatGPT as an AI system capable of processing language. Within the simulated scenario of submitting written materials, participants were instructed to consider collaborative creation of written content with ChatGPT, based on the needs of their work or study.

**Figure 4:**
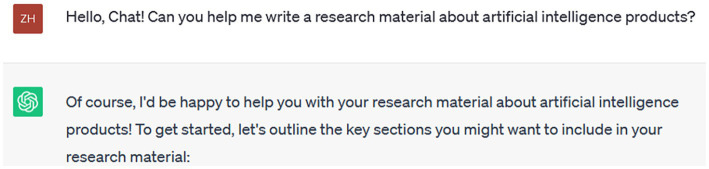
Artificial intelligence dominant of experiment 2.

**Figure 5:**
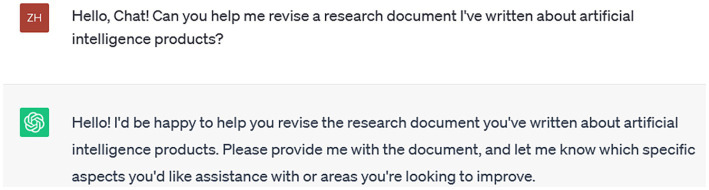
Artificial intelligence assisted of experiment 2.

According to the different collaboration types, participants were randomly assigned to two groups: AI-dominant group and AI-assisted group, and were presented with distinct experimental contexts. Group AI-D participants were presented with Figure 4, while Group AI-A participants were shown in Figure 5. In the AI-dominant group, participants were asked to imagine using ChatGPT to directly generate a piece of written content as needed. Conversely, in the AI-assisted group, participants were asked to imagine using ChatGPT to revise and refine their already completed written materials.

Upon completing the questions regarding their perception of collaboration types, participants from both groups were further randomly assigned to different outcome expectancy groups and were provided with the corresponding experimental descriptions. Participants in the positive outcome expectancy group were informed that their submitted written materials had received approval from superiors, deeming them as qualified. Conversely, participants in the negative outcome expectancy group were informed that their written materials had not passed the review of superiors and were rejected. Subsequently, participants were required to answer questions concerning their perception of the expected outcomes, aiming to provide researchers with insights into their feelings and attitudes toward the anticipated results.

After reading the experimental scenario, similar to Experiment 1, participants completed the evaluation items for ChatGPT and their willingness to use it. Subsequently, participants were required to complete three measurements of responsibility attribution (Peterson et al., 1982, 287–299; McAuley et al., 1992, 566–573): “I believe that this outcome is the responsibility (contribution) of ChatGPT,” “I feel that this outcome is largely due to ChatGPT,” and “I feel that this outcome is strongly related to ChatGPT.” Furthermore, considering that AI has been suggested to threaten consumers’ perceived control, subsequently affecting satisfaction and willingness to use AI products and services (Sundar, 2020, 74–88), three items were employed to assess participants’ perceived control in order to eliminate the potential mediating mechanism of perceived control. All of the aforementioned items were assessed using a 7-point Likert scale, with 1 indicating strongly disagree and 7 indicating strongly agree.

Upon completing the aforementioned experiments, demographic variables such as gender and age were also measured for the participants.

#### 4.3.3. Experimental results

(1) Manipulation check

The results of independent-samples t-tests revealed that participants in the AI-dominant group (M_AI-dominant_ = 6.012, SD = 0.875, *n* = 84) perceived the impact of AI significantly higher than those in the AI-assistant group (M_AI-assistant_ = 4.835, SD = 1.534, *n* = 85; *t* = 6.115, *p* < 0.001, Cohen’s *d* = 0.943), indicating the successful manipulation of the perception of human-AI collaboration type. Another set of independent-samples t-tests showed that participants in the positive outcome expectation group had a significantly higher perception of task success (M_positive_ = 6.11, SD = 0.730, *n* = 89) compared to the negative outcome expectation group (M_negative_ = 3.63, SD = 1.803, *n* = 80; *t* = 11.974, *p* < 0.001, Cohen’s *d* = 1.803), suggesting the effectiveness of outcome expectation manipulation.

(2) Hypothesis testing

Interaction effects re-examination: A two-way ANOVA revealed significant interaction effects of human-AI collaboration type and outcome expectation on consumer evaluation [*F*(1, 165) = 18.531, *p* < 0.001, η^2^ = 0.101] and usage intention [F(1, 165) = 15.795, *p* < 0.001, η^2^ = 0.087], confirming the validation of Hypothesis H1. Further analysis of simple effects indicated that under positive outcome expectation, the AI-dominant group showed higher evaluation [M_AI-dominant_ = 6.053, SD = 0.393 vs. M_AI-assistant_ = 5.800, SD = 0.494; F(1, 165) = 5.520, *p* < 0.05] and usage intention [MAI-dominant = 6.136, SD = 0.343 vs. MAI-assistant = 5.867, SD = 0.566; F(1, 165) = 8.380, *p* < 0.01] than the AI-assistant group, thus validating Hypothesis H1a. On the contrary, under negative outcome expectation, the AI-dominant group had lower evaluation [M_AI-dominant_ = 3.833, SD = 1.369 vs. M_AI-assistant_ = 4.850, SD = 1.247; F(1, 165) = 13.146, *p* < 0.001] and usage intention [M_AI-dominant_ = 4.086, SD = 1.532 vs. M_AI-assistant_ = 5.083, SD = 1.290; F(1, 165) = 9.550, *p* < 0.01] compared to the AI-assistant group, confirming Hypothesis H1b.

**Figure 6:**
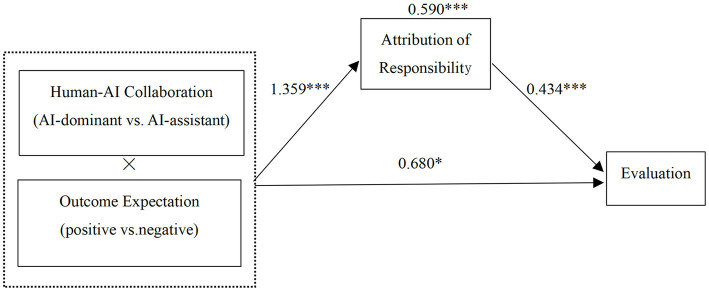
Mediating effect of responsibility attribution on consumer evaluation.

**Figure 7:**
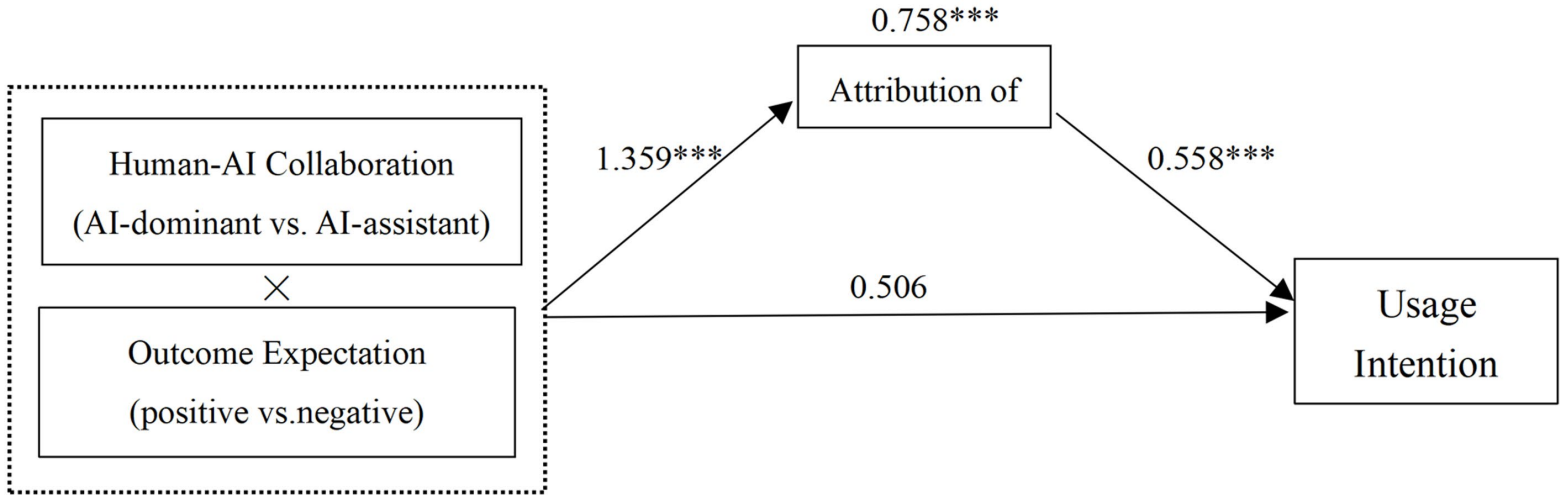
Mediating effect of responsibility attribution on consumer usage intention.

Mediation Analysis: Using Model 8 in the PROCESS program with 5,000 bootstrap samples and a 95% confidence interval, mediation effects of responsibility attribution were tested as mediators for consumer evaluation and usage intention (Hayes, 2013, 335–337). The results, as presented in Table 1, demonstrated that responsibility attribution played a mediating role in the interaction effects of human-AI collaboration type and outcome expectation on consumer evaluation (LLCI = 0.217, ULCI = 1.004, not including 0) and usage intention (LLCI = 0.332, ULCI = 1.211, not including 0), with mediation effects of 0.590 and 0.758, respectively. This confirms Hypothesis H2, as depicted in Figures 6, 7. Further analysis revealed that under negative outcome expectation, the mediation effect of responsibility attribution on consumer evaluation (LLCI = −0.705, ULCI = −0.147, not including 0) and usage intention (LLCI = −0.865, ULCI = −0.221, not including 0) was significant. Similarly, under positive outcome expectation, the mediation effect of responsibility attribution on consumer evaluation (LLCI = 0.056, ULCI = 0.244, not including 0) and usage intention (LLCI = 0.080, ULCI = 0.415, not including 0) was also significant. In summary, responsibility attribution serves as a mediator in the process of the interaction effects of human-AI collaboration type and outcome expectation on consumer evaluation and usage intention, confirming Hypothesis H2.

**Table 1:** Mediation analysis of responsibility attribution (Experiment 2).

Dependent variable	Effect type		Effect	SE	95% Confidence interval
Evaluation	Direct effect		0.680	2.089	[0.037, 1.323]
	Indirect effect		0.590	0.201	[0.217, 1.004]
		Negative outcome	−0.409	0.144	[−0.705, −0.147]
		Positive outcome	0.181	0.075	[0.056, 0.244]
Usage intention	Direct effect		0.506	0.345	[−0.175, 1.186]
	Indirect effect		0.758	0.225	[0.332, 0.189]
		Negative outcome	−0.525	0.165	[−0.865, −0.221]
		Positive outcome	0.233	0.086	[0.080, 0.415]

To verify the rationality of responsibility attribution as a mediating mechanism, it is essential to further exclude the potential mediating role of perceived control. This involves examining whether perceived control mediates the interaction effects of human-AI collaboration type and outcome expectation on consumer evaluation and usage intention. Using Model 8 in the PROCESS program with 5,000 bootstrap samples and a 95% confidence interval, mediation effects of perceived control were tested as mediators for consumer evaluation and usage intention (Hayes, 2013, 335–337), as shown in Table 1. The results indicated that perceived control did not mediate the interaction effects of human-AI collaboration type and outcome expectation on consumer evaluation (LLCI = −0.031, ULCI = 0.205, including 0) and usage intention (LLCI = −0.067, ULCI = 0.189, including 0), as demonstrated in Table 2. In conclusion, perceived control does not mediate the process of the interaction effects of human-AI collaboration type and outcome expectation on consumer evaluation and usage intention. Thus, the mediating role of perceived control is ruled out.

**Table 2:** Mediation analysis of perceived control (Experiment 2).

Dependent variable	Effect type		Effect	SE	95% Confidence interval
Evaluation	Direct effect		1.211	0.295	[0.629, 1.793]
	Indirect effect		0.059	0.062	[−0.031, 0.205]
		Negative outcome	0.018	0.044	[−0.061, 0.122]
		Positive outcome	0.076	0.058	[−0.026, 0.205]
Usage intention	Direct effect		1.221	0.320	[0.590, 1.852]
	Indirect effect		0.043	0.063	[−0.067, 0.189]
		Negative outcome	0.013	0.041	[−0.053, 0.121]
		Positive outcome	0.056	0.065	[−0.066, 0.192]

#### 4.3.4. Discussion of experiment 2

Experiment 2 examined the interaction effects of human-AI collaboration type and outcome expectation, as well as the mediating role of responsibility attribution. The results revealed that the interaction effects of human-AI collaboration type and outcome expectation persisted in AI generative products and services. Moreover, responsibility attribution mediated the interactive impact of human-AI collaboration type and outcome expectation on consumer evaluation and usage intention of AI products and services, confirming Hypothesis H2. Under positive outcome expectations, consumers displayed higher evaluation and usage intention toward AI-dominant products and services compared to AI-assistive ones, confirming Hypothesis H1a. Conversely, under negative outcome expectations, consumers exhibited higher evaluation and usage intention toward AI-assistive products and services compared to AI-dominant ones, validating Hypothesis H1b. The interaction effects of human-AI collaboration type and outcome expectation on consumer evaluation and usage intention for AI products and services remained evident.

### 4.4. Experiment 3

The aim of Experiment 3 was to investigate the moderating role of algorithm transparency in the interaction effects of human-AI collaboration type and outcome expectation. Additionally, this experiment aimed to reexamine the interaction effects and the mediating role, while introducing the factor of algorithm transparency.

#### 4.4.1. Design and participants

Experiment 3 employed a 2 × 2 × 2 between-subjects design, encompassing the factors of human-AI collaboration type (AI-dominant vs. AI-assistive), outcome expectation (positive vs. negative), and algorithm transparency (high vs. low). The experiment utilized a clinical decision-making system as the experimental material, focusing on exploring the current applications of AI in the medical domain.

In this experiment, a total of 386 participants were recruited through the Credamo platform, under the premise of assessing consumer evaluations and willingness to use prior to the introduction of a clinical decision-making system for medical diagnosis in a hospital. After excluding 33 questionnaires due to inconsistencies and inadequate responses, a total of 353 valid participants remained. Among them, 209 were females, accounting for 59.2%, and 144 were males, accounting for 40.8%. All participants provided informed consent, and the design was approved by the appropriate ethics review board. The age range of participants was between 18 and 60 years.

Participants were randomly assigned to eight different groups: AI-dominant/negative outcome/low algorithm transparency group (46 participants), AI-assistive/negative outcome/low algorithm transparency group (44 participants), AI-dominant/negative outcome/high algorithm transparency group (47 participants), AI-assistive/negative outcome/high algorithm transparency group (47 participants), AI-dominant/positive outcome/low algorithm transparency group (43 participants), AI-assistive/positive outcome/low algorithm transparency group (43 participants), AI-dominant/positive outcome/high algorithm transparency group (41 participants), and AI-assistive/positive outcome/high algorithm transparency group (40 participants).

#### 4.4.2. Experimental procedure

Firstly, participants are guided into a hypothetical scenario where they are undergoing a routine full-body examination at a renowned hospital known for its advanced medical equipment. Recently, the hospital has introduced a clinical decision-making system based on artificial intelligence technology. Once participants have familiarized themselves with this shared scenario, they are randomly assigned to either the high algorithm transparency group or the low algorithm transparency group.

In the high algorithm transparency group, participants receive approximately 280 words of detailed textual material explaining the functions, operational principles, and decision-making processes of the clinical decision-making system. Conversely, in the low algorithm transparency group, participants are provided with approximately 280 words describing the advantages of artificial intelligence technology and its application in certain domains. Subsequently, participants are required to answer two questions assessing algorithm transparency, adapted from Lehmann et al. (2022, 3419-3434): “I have a general understanding of how the clinical decision-making system works” and “I am aware that the clinical decision-making system may make errors” (using a 7-point rating scale, where 1 indicates strongly disagree and 7 indicates strongly agree).

Following the reading of the textual material, participants are further randomly assigned to either the AI-dominant group or the AI-assistive group, along with additional experimental materials describing different applications of the clinical decision-making system. In the AI-dominant group, participants learn that the clinical decision-making system independently diagnoses patient conditions. Conversely, in the AI-assistive group, participants learn that doctors utilize the clinical decision-making system to assist in diagnosing patient conditions. Subsequently, participants respond to questions related to their perception of human-AI collaboration type.

Subsequent to this, participants are once again divided into the positive outcome expectation group or the negative outcome expectation group based on the information they received. In the positive outcome expectation group, participants receive information that multiple patients’ conditions and causes were successfully diagnosed, leading to timely treatment. In the negative outcome expectation group, participants receive information about a case of misdiagnosis resulting in treatment delays. At this point, participants answer questions related to their perception of the expected outcomes.

After reading the experimental scenario, similar to the aforementioned study, participants are required to complete assessment items regarding the clinical decision-making system, willingness to use, and attribution of responsibility. Following the completion of the aforementioned experiment, demographic variables such as gender and age of the participants are measured.

#### 4.4.3. Experimental results

(1) Manipulation check

The results of the independent samples t-tests indicate that the participants in the AI-dominant group (M_AI-dominant_ = 6.249, SD = 0.796, *n* = 177) significantly perceived the impact of AI more positively than the participants in the AI-assistant group (M_AI-assistant_ = 4.296, SD = 1.720, *n* = 174; *t* = 13.683, *p* < 0.001, Cohen’s *d* = 1.457), confirming the successful manipulation of the human-AI collaboration type. Similarly, the participants in the positive outcome expectation group (M_positive_ = 5.99, SD = 0.776, *n* = 184) significantly perceived a higher level of task success than those in the negative outcome expectation group (M_negative_ = 3.41, SD = 1.644, *n* = 167; *t* = 18.500, *p* < 0.001, Cohen’s *d* = 2.007), indicating the effectiveness of the outcome expectation manipulation. Furthermore, the participants in the high algorithm transparency group (M_high transparency_ = 6.051, SD = 0.689, *n* = 176) demonstrated a significantly better understanding of the workings of the clinical decision-making system compared to the participants in the low algorithm transparency group (M_low transparency_ = 4.597, SD = 1.416, *n* = 175; *t* = 12.237, *p* < 0.001, Cohen’s *d* = 1.306), confirming the success of the algorithm transparency manipulation.

(2) Hypothesis testing

Interaction effects reexamination. The results of a two-way ANOVA revealed a significant interaction effect between human-AI collaboration type and outcome expectation on consumer evaluation [*F*(1, 349) = 35.717, *p* < 0.001, η^2^ = 0.093] as well as on usage intention [F(1, 349) = 31.634, *p* < 0.001, η^2^ = 0.084], thereby effectively confirming Hypothesis 1. Subsequent analyses of simple effects further elucidated that under positive outcome expectation conditions, the AI-dominant group exhibited higher evaluation scores [M_AI-dominant_ = 6.067, SD = 0.413 vs. M_AI-assistant_ = 5.767, SD = 0.595; F(1, 349) = 13.390, *p* < 0.001] and usage intention scores [M_AI-dominant_ = 6.032, SD = 0.529 vs. M_AI-assistant_ = 5.755, SD = 0.776; F(1, 349) = 7.131, *p* < 0.01] compared to the AI-assistant group, substantiating Hypothesis 1a. Similarly, during negative outcome expectation conditions, the AI-dominant group reported lower evaluation scores [M_AI-dominant_ = 3.953, SD = 1.603 vs. M_AI-assistant_ = 4.993, SD = 1.065; F(1, 349) = 22.708, *p* < 0.001] and usage intention scores [M_AI-dominant_ = 3.391, SD = 1.578 vs. M_AI assistant_ = 4.963, SD = 1.102; F(1, 349) = 20.572, *p* < 0.001] compared to the AI-assistant group, confirming Hypothesis 1b.

Mediation Effects Reexamination. Utilizing the PROCESS program’s Model 8 with 5,000 bootstrap samples and a 95% confidence interval, mediation effects of responsibility attribution were evaluated with consumer evaluation and usage intention as dependent variables (Hayes, 2013, 335–337). The specific results, as presented in Table 3, underscore that responsibility attribution mediated the influence of the interaction between human-AI collaboration type and outcome expectation on consumer evaluation (LLCI = 0.047, ULCI = 0.342, not including 0) and usage intention (LLCI = 0.006, ULCI = 0.321, not including 0), with mediation effects of 0.186 and 0.157 respectively, confirming Hypothesis 2. Further dissection of the results indicated that under negative outcome expectation, responsibility attribution significantly mediated consumer evaluation (LLCI = −0.170, ULCI = −0.019, not including 0) and usage intention (LLCI = −0.156, ULCI = −0.003, not including 0); similarly, under positive outcome expectation, responsibility attribution mediation effects were significant for consumer evaluation (LLCI = 0.024, ULCI = 0.196, not including 0) and usage intention (LLCI = 0.003, ULCI = 0.190, not including 0). In sum, responsibility attribution acted as a mediator in the process of the interaction between human-AI collaboration type and outcome expectation influencing consumer evaluation and usage intention, effectively confirming Hypothesis 2.

**Table 3:** Mediation analysis of responsibility attribution (Experiment 3).

Dependent variable	Effect type		Effect	SE	95% Confidence interval
Evaluation	Direct effect		1.154	0.232	[0.697, 1.611]
	Indirect effect		0.186	0.075	[0.047, 0.342]
		Negative outcome	−0.086	0.039	[−0.170, −0.019]
		Positive outcome	0.100	0.044	[0.024, 0.196]
Usage intention	Direct effect		1.151	0.242	[0.675, 1.627]
	Indirect effect		0.157	0.080	[0.006, 0.321]
		Negative outcome	−0.072	0.039	[−0.156, −0.003]
		Positive outcome	0.085	0.047	[0.003, 0.190]

**Figure 8:**
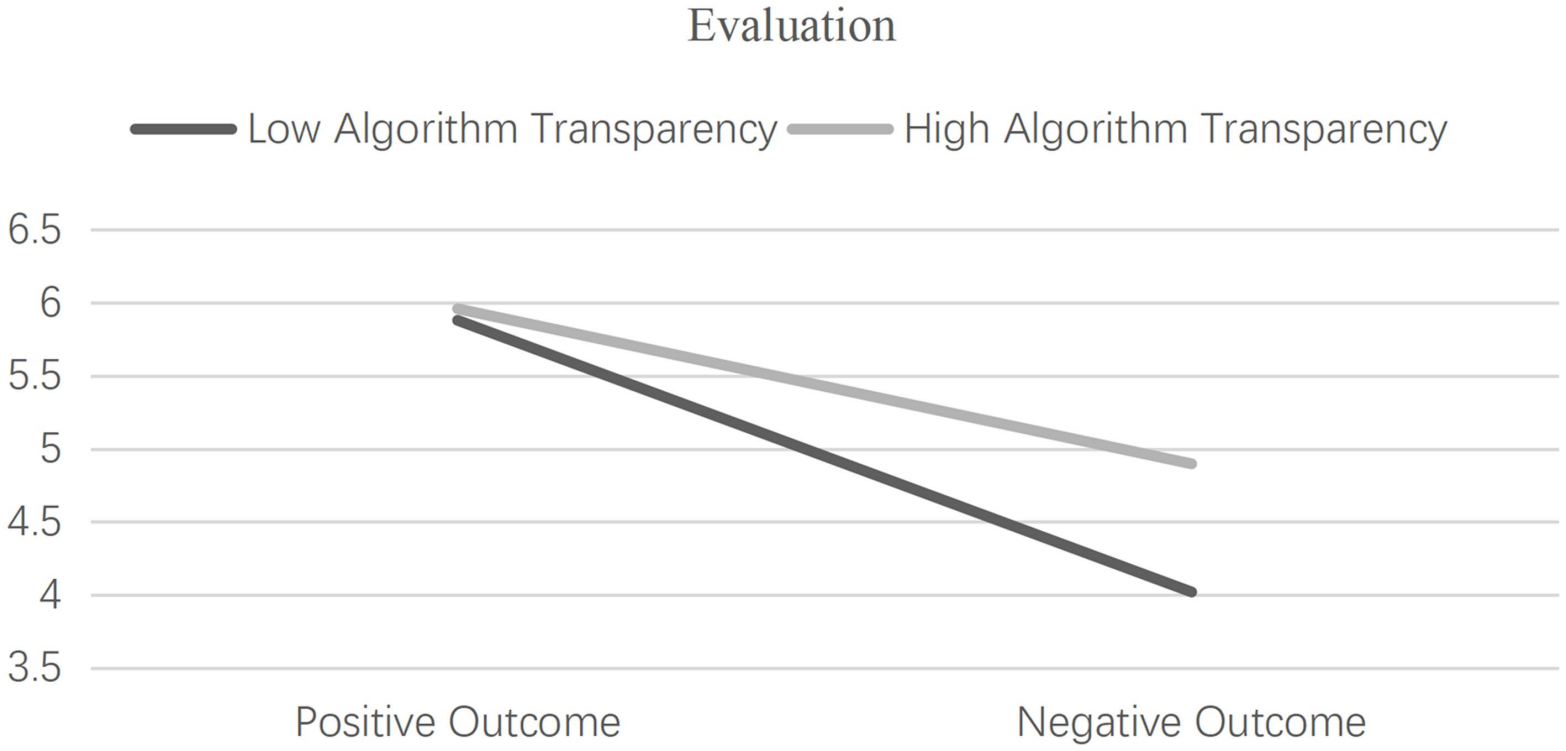
The moderating role of algorithm transparency (consumer evaluation).

**Figure 9:**
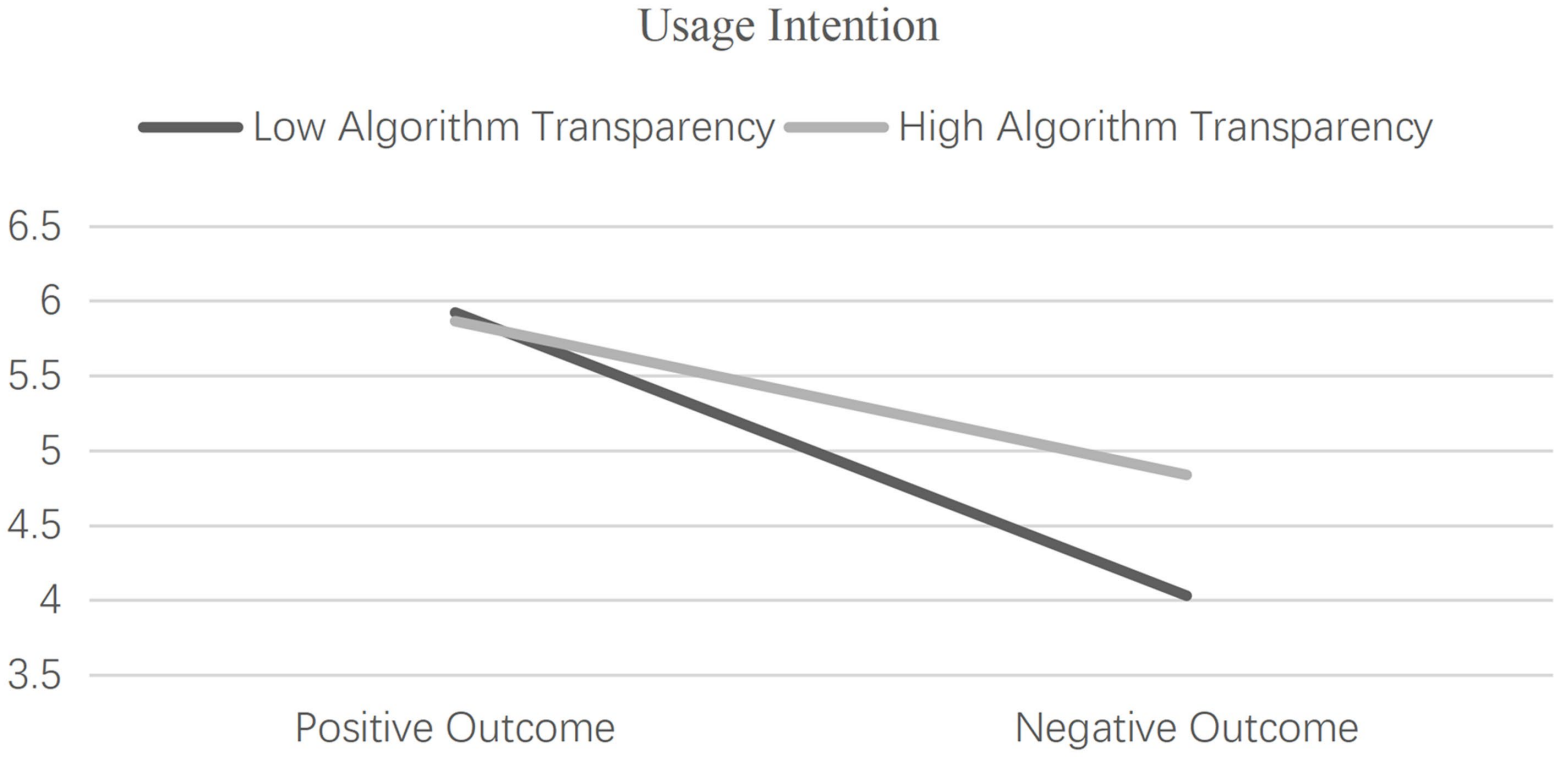
The moderating role of algorithm transparency (consumer usage intention).

Moderation Effects Examination. Utilizing separate univariate ANOVA tests with consumer evaluation and usage intention as dependent variables, the moderation effects of algorithm transparency were scrutinized. The results unveiled a significant three-way interaction effect of human-AI collaboration type, outcome expectation, and algorithm transparency on consumer evaluation [*F*(1, 352) = 41.699, *p* < 0.001, η^2^ = 0.460] as well as usage intention [F(1, 352) = 36.812, *p* < 0.001, η^2^ = 0.429], thus substantiating the moderating role of algorithm transparency in the interaction between human-AI collaboration type and outcome expectation, as illustrated in Figures 8, 9, and confirming Hypothesis 3. To further unveil the essence of the moderation effect by algorithm transparency, independent samples t-tests were conducted.

Under positive outcome expectation, no significant differences were observed between the high algorithm transparency group and the low algorithm transparency group in terms of their evaluation of AI products and services [M_high transparency_ = 5.959, SD = 0.470, *n* = 81 vs. M_low transparency_ = 5.880, SD = 0.585, *n* = 86; *t* = 0.959, *p* = 0.339, Cohen’s *d* = 0.149], as well as their usage intention [M_high transparency_ = 5.864, SD = 0.605, *n* = 81 vs. M_low transparency_ = 5.922, SD = 0.739, *n* = 86; *t* = −0.556, *p* = 0.579, Cohen’s d = −0.086], confirming Hypothesis 3a. Conversely, under negative outcome expectation, the high algorithm transparency group demonstrated significantly higher evaluation scores [M_high transparency_ = 4.897, SD = 1.248, n = 94 vs. M_low transparency_ = 4.019, SD = 1.527, *n* = 90; *t* = 4.281, *p* < 0.001, Cohen’s *d* = 0.630] and usage intention scores [M_high transparency_ = 4.837, SD = 1.329, *n* = 94 vs. M_low transparency_ = 4.030, SD = 1.473, *n* = 90; *t* = 3.907, *p* < 0.001, Cohen’s *d* = 0.575] compared to the low algorithm transparency group, thus validating Hypothesis 3b.

#### 4.4.4. Discussion of experiment 3

Experiment 3 delved into the interactive effects of human-AI collaboration type and outcome expectation on consumer evaluation and usage intention toward AI products and services, the mediating role of responsibility attribution, and the moderating influence of algorithm transparency. The results unveiled distinctive patterns. Under positive outcome expectation, consumers were inclined to attribute higher success contributions to AI-dominant collaboration compared to AI-assisted collaboration, resulting in elevated consumer evaluation and usage intention toward AI-dominant products and services. Conversely, in situations of negative outcome expectation, consumers were more prone to attribute failure responsibility to the AI technology within AI-dominant collaboration, consequently leading to reduced usage intention toward AI-dominant products and services in comparison to AI-assisted ones. Thus, Hypotheses 1 and 2 were validated. Additionally, the moderating role of algorithm transparency was substantiated as it demonstrated a more pronounced effect in adjusting consumer evaluation and usage intention toward AI products and services under negative outcome expectation, validating Hypothesis 3.

## 5. Conclusion

### 5.1. Research findings

In addressing the differential consumer usage intention toward AI products and services, this study offers an explanatory perspective from the realm of human-AI collaboration through three experiments. Guided by the Technology Acceptance Model and Prospect Theory, this research investigates the interaction effect of human-AI collaboration type and outcome expectation on consumer evaluation and usage intention toward AI products and services, while also delving into the mediating role of responsibility attribution in the context of this interaction. Furthermore, the study explores the moderating role of algorithm transparency in the interaction effect between human-AI collaboration type and outcome expectation.

1. Human-AI collaboration type and outcome expectation interactively influence consumer evaluation and usage intention toward AI products and services. Specifically, under positive outcome expectation, consumers tend to affirm the positive role of AI technology, and due to the greater impact of AI technology in AI-dominant products and services compared to AI-assisted ones, consumers exhibit higher evaluation and usage intention toward AI-dominant products and services. Conversely, in situations of negative outcome expectation, consumers perceive AI technology as a source of uncertainty and risk, leading to lower evaluation and usage intention toward AI-dominant products and services compared to AI-assisted ones.
2. Responsibility attribution mediates the interaction effect of human-AI collaboration type and outcome expectation on AI products and services. In the context of positive outcome expectation, owing to the higher influence of AI in AI-dominant products and services relative to AI-assisted ones, consumers attribute greater contributions to AI-dominant products and services, resulting in higher evaluation and usage intention toward AI-dominant ones. However, under negative outcome expectation, consumers tend to attribute failure to AI, particularly in AI-dominant products and services with a higher impact of AI, leading to lower evaluation and usage intention toward AI-dominant products and services compared to AI-assisted ones.
3. Algorithm transparency moderates the interaction effect of human-AI collaboration type and outcome expectation. Specifically, higher algorithm transparency enhances consumers’ understanding of AI’s decision-making process and operational principles. Under negative outcome expectation, higher algorithm transparency boosts consumer evaluation and usage intention toward AI products and services. On the other hand, under positive outcome expectation, regardless of consumers’ comprehension of AI decision-making processes and operational principles, the positive halo effect of favorable outcomes contributes to positive evaluation and usage intention toward both AI-dominant and AI-assisted products and services.

### 5.2. Theoretical contribution

This study employed psychological theories such as the Technology Acceptance Model, Prospect Theory, and Attribution Theory, contributing to the enrichment and extension of these theories in the field of artificial intelligence. It provides a profound understanding of consumer emotions and decision-making processes, particularly in the context of differential responses to positive and negative outcome expectations. In summary, within the context of the rapid advancement of AI, this study attempts to address three thought-provoking questions for humanity: “Under what circumstances are humans willing to accept the dominant role of AI in human-AI collaboration?” “Why are humans willing or unwilling to accept the dominant role of AI in human-AI collaboration?” “How can the acceptance of AI by humans be enhanced?”

By investigating the mutual influence of human-intelligence collaboration types and outcome expectations, this study extends the theory of human-intelligence collaboration relationships within the consumer domain, offering a novel perspective for further research on the interaction between humans and AI. Currently, research on variations in the acceptance of AI products and services remains largely grounded in the perception of artificial intelligence as a mere tool, often overlooking a redefined understanding of AI’s role and the dynamics of human-AI relationships (Rudin and Carlson, 2019, 44–72). Building upon the exploration of human-AI relationships, this study introduces an innovative categorization of human-AI collaboration into AI-dominant and AI-assisted types. It subsequently delves into the interactive influence of different human-AI collaboration types and outcome expectation on the differentiated evaluation and usage intention of AI products. The findings highlight that consumers are more susceptible to variations in evaluation and usage intention toward AI-dominant products compared to AI-assisted ones, particularly under the influence of outcome expectation.

Furthermore, by considering responsibility attribution as an intermediary mechanism, this study highlights the issue of responsibility allocation in human-AI collaboration, which is crucial for explaining consumer attitudes toward AI products and services. This research posits that consumer evaluation and usage intention of AI products signify the degree of acceptance toward the AI entity, necessitating an examination of the attribution of responsibilities between human and AI agents during the collaborative process. Prior studies addressing attribution often focused on negative contexts, overlooking successful attributions in positive result scenarios (van der Woerdt and Haselager, 2019, 93–100). Thus, this study comprehensively demonstrates the mediating effect of responsibility attribution, unraveling the differential attributions made by consumers in distinct human-AI collaboration types under varying result expectancies, subsequently affecting the degree of acceptance of AI products and services. The results indicate that attributions toward AI are more pronounced in AI-dominant human-AI collaborations, leading to variations in evaluation and usage intention under different result expectancies.

Lastly, this study attempts to address the question of how to improve consumer attitudes toward AI products and services by introducing the technological feature of algorithm transparency, thereby enriching the research on AI in the consumer domain. Despite the prevalent concern over the “black-box” nature of algorithms (Ribeiro et al., 2016, 1,135–1,144), the marketing domain has given comparatively less attention to the impact of algorithm transparency on the differentiated evaluation and usage intention of AI products. The findings reveal that algorithm transparency enhances consumers’ understanding of AI decision-making processes and fosters trust in AI, particularly magnifying positive evaluation and usage intention of AI products under negative result expectancies. This contributes to a deeper understanding of the role of algorithm transparency in the context of marketing research.

### 5.3. Management insights

First and foremost, companies in the field of intelligent technology need to recognize the significance of positive outcome expectation in shaping consumers’ attitudes and acceptance toward AI products and services. Research demonstrates that under positive outcome expectation, consumers exhibit higher evaluation and usage intention toward AI-dominant products and services compared to AI-assisted ones. Conversely, in situations of negative outcome expectation, consumers tend to question AI technology and display low acceptance levels, particularly toward AI-dominant products. On one hand, intelligent technology companies should proactively communicate the positive potential and anticipated outcomes of AI products and services, striving to establish favorable product reputations that enhance consumers’ positive expectations. On the other hand, companies should continually enhance and refine the performance of AI technology, ensuring excellence in AI product functionality and service quality.

Secondly, companies should emphasize product design and user experience. Consumers attribute differently based on various result expectancies, which impacts their usage intention toward AI products and services. Under positive outcome expectation, consumers attribute greater success to AI-dominant products and services compared to AI-assisted ones, resulting in a higher inclination toward AI-dominance. Conversely, in scenarios of negative outcome expectation, consumers attribute negative outcomes more to AI-dominant products and services, leading to diminished usage intention. Consequently, there are elevated expectations for AI-dominant products in the market. When developing AI products, companies should focus on providing user-friendly, efficient, and reliable AI solutions to ensure consumers achieve positive outcomes and satisfactory experiences. Additionally, highlighting the role of AI as an assisting agent can help mitigate negative attributions toward dominant AI products.

Lastly, managers should strive to establish transparent and trustworthy AI systems. Consumer skepticism toward AI technology often arises from a lack of understanding regarding its functioning principles, algorithms, and concerns related to data privacy and security (Zarifis et al., 2021, 66–83). Research indicates that increasing algorithm transparency aids consumers in comprehending the algorithmic logic behind AI products and services, thereby enhancing evaluation and usage intention of AI products under negative outcome expectation. Therefore, companies should not only dedicate efforts toward enhancing and perfecting AI technology but also concurrently focus on improving consumers’ understanding of AI decision-making processes. Explaining the workings, algorithms, and data usage methods of AI products, addressing consumer doubts and inquiries, can alleviate the impact of negative outcome expectation on AI technology, thereby elevating consumer usage intention toward products and services (Laato et al., 2022, 1–31).

### 5.4. Limitations and prospects

While this study endeavors to elucidate variations in consumers’ willingness to use AI products and services, certain limitations remain. Firstly, differentiation in consumers’ attitudes toward AI products and usage intention might also be influenced by other contextual factors, such as task allocation (Karray et al., 2008, 137–159), user roles, and task importance, all of which could impact the willingness to adopt AI technology (Kaur and Rampersad, 2018, 87–96). Secondly, this study introduces responsibility attribution as an intermediary mechanism within the interaction effects of human-AI collaboration types and result expectancies. In the future, it is imperative to consider the influence of variables like perceived AI capability (Cuddy et al., 2008, 61–149) and algorithm aversion (Kim et al., 2019, 1–12). Thirdly, this research solely contemplates the scenario of low AI algorithm transparency at the current stage. The future proliferation of artificial intelligence knowledge could mitigate the “black-box” issue’s impact, necessitating further exploration of the moderating role of consumer individual traits, such as technological confidence and tolerance. Fourth, while we aimed to validate the generalizability of our findings through three distinct experiments, the variability in experimental contexts may be susceptible to different sources of external noise and variables. Lastly, addressing the variations in consumers’ evaluations and usage intentions toward AI products and services, future studies could contemplate employing qualitative research methods for in-depth exploration.

## Data availability statement

The raw data supporting the conclusions of this article will be made available from the corresponding author upon reasonable request.

## Ethics statement

The studies involving humans were approved by Academic Committee of the School of Business, Qingdao University, affiliated with Qingdao University. The studies were conducted in accordance with the local legislation and institutional requirements. These studies were conducted with the online informed consent of the participants.

## Author contributions

BY: Formal analysis, Methodology, Resources, Supervision, Validation, Visualization, Writing – review & editing, Investigation, Writing – original draft. HL: Conceptualization, Data curation, Formal analysis, Investigation, Methodology, Software, Writing – original draft, Writing – review & editing.
